# Actinomycetes for Marine Drug Discovery Isolated from Mangrove Soils and Plants in China

**DOI:** 10.3390/md7010024

**Published:** 2009-02-03

**Authors:** Kui Hong, An-Hui Gao, Qing-Yi Xie, Hao Gao, Ling Zhuang, Hai-Peng Lin, Hai-Ping Yu, Jia Li, Xin-Sheng Yao, Michael Goodfellow, Ji-Sheng Ruan

**Affiliations:** 1 Institute of Tropical Bioscience and Biotechnology, Chinese Academy of Tropical Agriculture Sciences, Haikou 571101, P.R.China;; xie-qy@sohu.com; linhp010612@gmail.com; 2 National Center for Drug Screening, Shanghai Institute of Materia Medica, Shanghai 201203, China E-mails: ahgao@mail.shcnc.ac.cn; hpyu@mail.shcnc.ac.cn; jli@mail.shcnc.ac.cn; 3 Institute of Traditional Chinese Medicine & Natural Products, College of Pharmacy, Jinan University, Guangzhou 510632, P.R. China; E-mail: ghao@mail.tsinghua.edu.cn, tyaoxs@jnu.edu.cn; 4 University of Newcastle, Newcastle upon Tyne NE1 7RU, UK; E-mail: m.goodfellow@ncl.ac.uk; 5 Institute of Microbiology, Chinese Academy of Sciences, Beijing, 100081, P.R. China; E-mail: jishengruan@yahoo.com.cn

**Keywords:** Mangroves, actinomycete diversity, marine drug discovery, high-throughput screening, growth inhibition, protein tyrosine phosphatase 1B, aurora kinase A, caspase 3

## Abstract

The mangrove ecosystem is a largely unexplored source for actinomycetes with the potential to produce biologically active secondary metabolites. Consequently, we set out to isolate, characterize and screen actinomycetes from soil and plant material collected from eight mangrove sites in China. Over 2,000 actinomycetes were isolated and of these approximately 20%, 5%, and 10% inhibited the growth of Human Colon Tumor 116 cells, *Candida albicans* and *Staphylococcus aureus*, respectively, while 3% inhibited protein tyrosine phosphatase 1B (PTP1B), a protein related to diabetes. In addition, nine isolates inhibited aurora kinase A, an anti-cancer related protein, and three inhibited caspase 3, a protein related to neurodegenerative diseases. Representative bioactive isolates were characterized using genotypic and phenotypic procedures and classified to thirteen genera, notably to the genera *Micromonospora* and *Streptomyces*. Actinomycetes showing cytotoxic activity were assigned to seven genera whereas only *Micromonospora* and *Streptomyces* strains showed anti-PTP1B activity. We conclude that actinomycetes isolated from mangrove habitats are a potentially rich source for the discovery of anti-infection and anti-tumor compounds, and of agents for treating neurodegenerative diseases and diabetes.

## 1. Introduction

It is indisputable that new drugs, notably antibiotics, are urgently needed to halt and reverse the relentless spread of antibiotic resistant pathogens which cause life threatening infections and risk undermining the viability of healthcare systems [1]. Filamentous bacteria belonging to the order *Actinomycetales*, especially *Micromomospora* and *Streptomyces* strains, have a unique and proven capacity to produce novel antibiotics [2–4], hence the continued interest in screening such organisms for new bioactive metabolites [5, 6]. However, it is becoming increasingly difficult to discover commercially significant secondary metabolites from well known actinomycetes as this practice leads to the wasteful rediscovery of known bioactive compounds, thereby emphasizing the need to isolate, characterize and screen reperesentatives of undiscovered actinomycete taxa. It is also becoming increasingly clear that un- and under-explored habitats, such as desert biomes and marine ecosystems, are a rich source of novel actinomycetes which have the capacity to produce interesting new bioactive compounds, including antibiotics [7–10].

Molecular ecological studies on community DNA extracted from deep-sea sediments revealed the presence of an astonishingly rich diversity of actinomycete taxa, most of which were predicted to represent novel species, genera and families [11, 12]. It is not surprising, therefore, that new species of known actinomycete genera isolated from marine habitats are being described on a regular basis [13–18]. There is also a steady stream of proposals for the recognition of new genera, as exemplified by the isolation of the *Demequina* from tidal mud flats [19], *Serinicoccus* from surface sea water [20] and *Salinispora* from oceanic sediments [21]. Representatives of these genera have an obligate requirement for salt, as do deep sea polar strains of *Dietzia* and *Rhodococcus* [22]. It has also been shown that nearly 60% of actinomycetes isolated from sediment samples collected from around the island of Guam in the Pacific Ocean required seawater for growth [23].

It is perhaps not surprising that novel marine actinomycetes are proving to be such a valuable source of new bioactive compounds [24–26] as actinomycete systematics is providing a taxonomic road map to genes hence products, including the discovery of first-in-class drug candidates [9, 27–29]. Indeed, an encouraging flow of novel anti-infection and anti-cancer compounds are being sourced from marine actinomycetes, as exemplified by the discovery of the abbysomicins, potent polycyclic polyketides active against methicillin-resistant *Staphylococcus aureus* and produced by “*Verrucosispora maris*” [30] and salinosporamide A, an anti-cancer compound produced by *Salinispora tropica* [31]. Marine *Verrucosispora* strains are also a source of novel proximicins, anti-tumor furan analogues of the antibiotic netropsin [32].

The discovery of novel microbial natural products is encouraged not only by the quality of biological material but also by the novelty of screening models. Many new molecular targets have been designed to detect anti-microbial and cytotoxic activities [33], and to highlight chemical entities for the treatment of conditions such as diabetes and degenerative diseases. Caspase 3, a key protease  involved in programmed cell death of neuronal apoptosis, for instance, is a promising target for the treatment of neurodegenerative diseases [34]. Similarly, potent and selective protein tyrosine phosphatase 1B inhibitors are potential therapy for the treatment of type-2 diabetes and obesity [35–37].

Mangroves, unique woody plant communities of intertidal coasts in tropical and subtropical coastal regions, are highly productive ecosystems [38, 39] though surprisingly little is known about the microbial communities living therein [40–42], although there is evidence that mangrove sediments contain high populations of micromonosporae [43] and novel actinomycetes, as illustrated by the isolation of *Asanoa iriomotensis* [44] and *Nonomuraea maheshkhaliensis* [13]. It is also encouraging that bioactive compounds have been obtained from mangrove plants [44–46], fungi [47–50], and bacteria [51], including actinomycetes [14, 52].

During 2001–2005, “China Sea” and “microorganism” showed the highest percentage of source region and source phyla citations, respectively, with respect to the total of marine natural products for 1965–2005 [24]. The rich mangrove flora of South-East China is composed of 26 plant species which are classified into 15 genera and 12 families [39]. All but one of the plant species, including four endemic ones, are found in mangroves located around the coast of Hainan Island. The primary aim of the present study was to determine whether actinomycetes isolated from environmental samples collected from mangrove forests in Fujian, Guangdong, Guangxi and Hainan Provinces in China showed biological activities. To this end, a range of selective isolation and characterization procedures were used to recover and identify diverse actinomycetes from plant and soil samples prior to establishing their activities in a number of biological screening assays. Representative isolates found to be active against tumor cells and a diabetes related protein were fully characterized using established chemotaxonomic, morphological and molecular systematic methods.

## 2. Results

### 2.1 Selective isolation of actinomycetes

A total of 2,041 actinomycetes were isolated from 112 soil and 99 plant samples collected from the eight mangrove sites, the average number of isolates per sample was 9.7 (2,041 from 211) (Table 1). The highest average number of actinomycetes, 21.8 (1,004 from 46), was obtained from the rhizosphere soil samples, and the lowest number, 4.5 (434 from 99), from the plant tissues samples. It is apparent that the number of endophytic actinomycetes isolated from plant tissues is much less than those recovered from the composite soil samples. The highest average number of isolates were obtained from the composite soil samples collected from the Fujian mangrove (93 isolates from 3 samples), and the lowest number from the corresponding Guangxi sample (62 isolates from 17 samples). In total, 603 actinomycetes were isolated from the composite soil samples giving an average nearly of 9.3 strains per sample.

### 2.2 Bioactivity of strains isolated from the mangrove sites

All of the isolates were examined to determine their activities in five high throughput screening models, namely against Human Colon Tumor (HCT) 116 cells, *Candida albicans* ATCC 10231, *Staphylococcus aureus* ATCC 51650, protein tyrosine phosphatase (PTP1B) and caspase 3. Three hundred and forty three strains showed activity against the tumor cell line (16.8%), 198 against *S. aureus* (9.7%)*,* 101 against *C. albicans* (4.9%), and 59 against PTP1B (2.9%); only 3 isolates inhibited caspase 3. In addition, 9 out of the 343 cytotoxic strains showed activity against aurora kinase A, a protein related to an indicator of anti-cancer activity. Some of the isolates exhibited activities in more than one of the screens.

**Figure 1:**
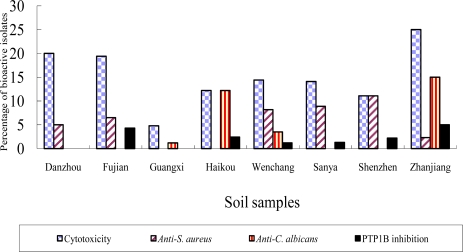
Percentage of bioactive strains isolated from composite soil samples collected from the mangrove sites. The percentages indicate the number of positive hits against each of 4 targets over the total number of strains isolated at each site.

The highest percentage of anti-tumor and anti- *C. albicans* activity was found with strains isolated from samples collected from Zhanjiang. Isolates from the Wenchang samples showed activity against  all of the screening models, including two out of the three isolates inhibit on caspase 3, possibly reflecting the diverse nature of the plant species growing at these well preserved locations (Figure 1). In contrast, the lowest number of bioactive strains was recorded from the Guangxi samples; it was noted that the soil from this location contained more sand and less organic matter than those from other sites.

It can be seen from Table 2 that a higher percentage of bioactive strains were isolated from the plant tissues compared with those isolated from the corresponding rhizosphere soil samples with respect strains isolated from the Wenchang samples but with two exceptions of cytotoxicity and PTP1B inhibition from the Zhangjiang samples.

### 2.3 Bioactivity of actinomycetes isolated from the 23 mangrove plant species

Higher total number of bioactive actinomycetes was obtained from the rhizosphere soil than from plant materials collected from the 23 mangrove plant species with the exception of anti-*C. albicans*. Strains isolated from either the rhizosphere soil or the plant tissues of *Acanthus ilicifolius* showed activity in all of the assays, apart from caspase 3.

The highest number of bioactive strains was observed from the rhizosphere soil of *Heritiera littoralis*. High numbers of bioactive strains were found in all of the assays, apart from caspase 3, in the case of isolates from the rhizosphere soil of *Heritiera littoralis,* and from the plant tissues of *Bruguiera gymnorrhiza*. In contrast, bioactive actinomycetes were not obtained from the rhizosphere soil or the plant tissues of *Sonneratia hainanensis*, an endemic plant in Hainan mangrove forests (Table 3).

### 2.4 Taxonomic diversity of bioactive actinomycetes

Two hundred and thirty seven isolates selected on the basis of their metabolites bioactivities were identified using chemotaxonomic (presence of isomers of diaminopimelic acid and diagnostic whole-organism sugars) and morphological (as seen by light microscopy) markers, and by 16S rRNA gene sequence data. The results showed that the tested isolates belonged to 13 genera classified into 7 families and 5 suborders (Table 4). It is clear from the table that the highest activities were shown by isolates assigned to the genera *Micromonospora* and *Streptomyces.* However, it is also interesting that the *Actinomadura, Nocardia* and *Nonomuraea* isolates showed anti-tumor cell activity.

### 2.5 16S rRNA gene sequencing of representative isolates showing activity against tumor cells

**Figure 2:**
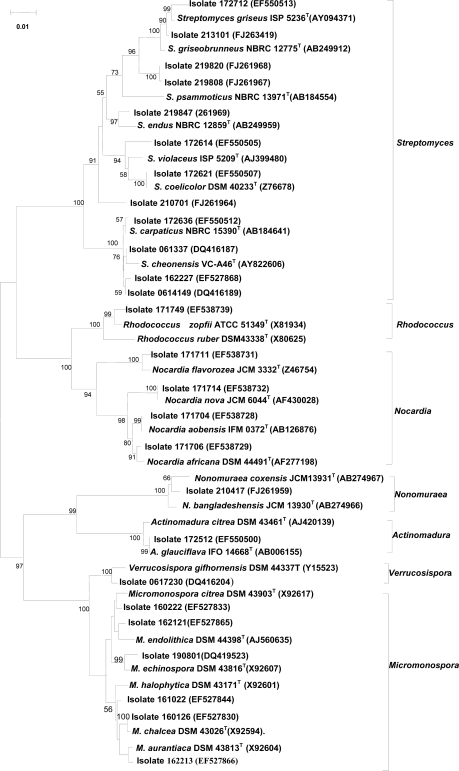
Neighbor-joining tree based on almost complete 16S rRNA gene sequences showing relationships between the 26 selected actinomycetes found to inhibit tumor cells *in vitro,* and between them and the type strains of the highest 16S rDNA sequence similarity. Numbers at the nodes indicate bootstrap values based on 1000 replicates; only values above 50% are shown. Bar, 1% sequence divergence.

The most taxonomically diverse actinomycetes were those showed activity against the tumor cells. It can be seen from Figure 2 that the 26 tested strains which gave positive results were assigned to 7 genera with just over half of them belonging to the genus *Streptomyces*. Most of the *Streptomyces* isolates formed distinct phyletic lines though strain 172621 was found to have an identical sequence to that of the type strain of *S. coelicolor*. Similarly, the 6 isolates belonging to the genus *Micromonospora* formed a diverse group, as did the 4 *Nocardia* isolates. It was interesting that the single isolate assigned to the genus *Actinomadura* had an identical 16S rRNA gene sequence to the type strain of *A. glauciflava*. In contrast, the single isolates belonging to the genera *Nonomuraea*, *Rhodococcus*, and *Verrucosispora* formed relatively distinct phyletic lines.

Some of the *Streptomyces* isolates were assigned to 16S rRNA subclades which reflected the sites from which they were isolated. In particular, the three strains from Wenchang, isolates 061337, 0614149 and 162227, formed a distinct subclade together with the type strain of *S. cheonensis*, a taxon supported by a 100% bootstrap value. Similarly, isolates 172614 and 172621 from the Shenzhen composite soil constituted a well delineated subclade together with the type strains of *S. coelicolor* and *S. violaceus.* The four strains isolated from the Sanya composite soil sample were part of a more diffuse subclade which encompassed the type strains of *S. endus, S. griseobrunneus, S. griseus* and *S. psammoticus* (Figure 2). Most of the *Micromonospora* strains which showed activity against tumor cells were isolated from the roots of mangrove plants, i.e., *Acrostichum aureum* (isolate160126), *Acrostichum speciosum* (isolate 160222), *Cerbera manghas* (isolate 162121), *Lumnitzera racemosa* (isolate 161022) and *Rhizophora stylosa* (162223). More micromonosporae than streptomycetes were isolated from the plant material.

### 2.6 16S rRNA gene sequences of isolates which inhibited protein tyrosine phosphatase 1B

**Figure 3:**
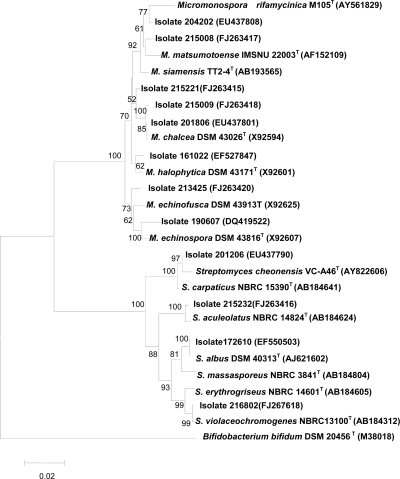
Neighbor-joining tree based on nearly complete 16S rRNA gene sequences showing relationships between the 12 isolates active in the PIP1B assay and between them and the highest 16S rDNA sequence similar *Micromonospora* and *Streptomyces* species. *Bifidobacterium bifidum* DSM 20456 ^T^ (M38018) was used as the outgroup. Numbers at the nodes are percentage bootstrap values based on 1,000 replicates; only values 50% or above are given. Bar, 2% sequence divergence.

The 12 actinomycetes those metabolites active in the PTP1B screen were assigned to the genera *Micromonospora* and *Streptomyces* (Figure 3). Six out of the 8 *Micromonospora* strains formed relatively distinct phyletic lines though isolates 201806 and 215009 had identical 16S rRNA gene sequences to one another and to the type strain of *M. chalcea*. The 4 *Streptomyces* isolates were relatively closely related to the type strains of *Streptomyces* species. Indeed, isolate 172610 had an identical 16S rRNA gene sequence to the type strain of *S. albus* whereas isolate 216802 had a nearly identical sequence to *S. violaceochromogenes* NBRC 13100^T^.

## 3. Discussion

Considerable attention is currently being paid to the isolation and characterization of novel actinomycetes from poorly researched habitats given the premise that screening such organisms raises the prospect of discovering new natural products that can be developed as a resource for biotechnology [7, 10, 43]. This reasoning appears to be sound as novel actinomycetes isolated from unexplored marine habitats are proving to be a valuable source of new bioactive metabolites [9, 26]. It seems timely, therefore, to extend this approach to another poorly studied environment, the mangrove ecosystem. Mangroves develop along estuaries in tropical and subtropical regions where sea water and fresh water mix. Indeed, mangrove forests may become an invaluable source for discovering novel actinomycetes hence new chemodiversity should salinity prove to be a driver of bacterial diversity as proposed by Lozupone and Knight [53].

It was not intended in the present investigation to assemble a detailed inventory of the kinds of actinomycetes found in the mangrove ecosystem or to isolate and characterize novel bioactive compounds, but the study was designed to prepare the ground for such studies by isolating, partially characterizing and screening a diverse range of actinomycetes. Even so, this investigation does provide the most comprehensive survey of culturable actinomycetes present in mangrove forests, a result which probably reflects the application of a broad range of selective isolation procedures to many environmental samples.

In total, representatives of 13 established actinomycete genera belonging to 7 families and 5 suborders were recognized out of the bioactive isolates. The presence of predominant numbers of *Micromonospora* and *Streptomyces* strains amongst the isolates is in line with the results reported by Eccleston *et al.* [43]. *Asanoa* and *Gordonia* can be added to the list of genera isolated from mangroves though the representatives of these taxa did not show any biological activity from the present screening, thus provides further evidence that such organisms are present in the mangrove ecosystem [13, 18].

Many of the strains isolated in the present study showed bioactivity against one or more of the screening systems, thereby underpinning and extending results from previous studies which showed mangroves to be a source of bioactive actinomycetes [14, 43]. Thus, 343 isolates showed activity against the tumor cell line (16.8%), 101 against *C. albicans* ATCC 10231(4.9%), 198 against *S. aureus* ATCC 51650 (9.7%)*,* 59 against PTP1B (2.9%); but only 3 and 9 strains were active against caspase 3, and aurora kinase A, respectively. The lower inhibition frequencies of biochemical assays on molecular level comparing to growth inhibition assays on cell level are acceptable considering their higher  selectivity. The most active strains belonged to the genera *Micromonospora* and *Streptomyces.* Indeed, members of these taxa showed activity against caspase 3 and protein tyrosine phosphatase 1B. In contrast, representatives of seven genera, namely *Actinomadura, Micromonospora, Nocardia, Nonomuraea, Rhodococcus, Streptomyces* and *Verrucosispora,* were active against the tumor cell line.

The distribution of bioactive actinomycetes showed some relationship to the nature and source of the environmental samples. The highest percentage of isolates showing anti-tumor and anti- *C. albicans* activity, for example, were from the Zhangjiang mangrove. Isolates from this site and from the mangrove at Wenchang were particularly effective in the anti-infective and anti-cancer assays. Similarly, higher numbers of bioactive strains were obtained from rhizosphere samples than from corresponding plant materials. The rhizosphere soil from *Heritiera littoralis* was a particularly rich source of bioactive strains. These results are not only interesting but will also guide future bioprospecting strategies.

It is becoming increasingly evident that the taxonomic and metabolic diversity encompassed by streptomycetes is remarkable, as new and putatively novel *Streptomyces* species are being continuously isolated from under-researched habitats and shown to be valuable sources of new bioactive compounds [8,10,26]. The 16S rRNA gene sequence data acquired in the present study provided further evidence of this trend. Thus many of the streptomycetes which showed activity in the anti-tumor assay can be assigned to new species as they are separated from the type strains phylogenetic neighbors by sequence similarities well below those found between closely related *Streptomyces* species, such as those classified in the *S. violaceusniger* subclades [27, 28] and *S. griseus* [54]. The highest 16S rDNA sequence similarity to the valid species of *Streptomyce violaeus* of isolate 172614 and isolate 210701 were lower than 0.97 as 0.95 and 0.968, respectively. Though the highest 16S rDNA sequence similarity of isolates of 219820 and 219808 to the type strain of *S. griseobrunneus* NBRC 12775^T^ were a little higher than 0.97, with the values of 0.977 and 0.976, respectively, they were highly lower than the similarity of most of the known closely related streptomycetes species [54]. Isolates 162227 and 0614149 formed a distinct branch with the highest 16S rDNA sequence similarity of 0.985 and 0.99, respectively, to the type strain of *S. carpaticus* NBRC15390^T^, but confirmed to be a new species by the DNA-DNA pairing value lower than 70% (data not shown). It is also interesting that some of the *Streptomyces* isolates were assigned to 16S rRNA subclades which corresponded to the sites from which the strains were isolated. These observations while tentative might reflect an endemic and /or environmental selection of *Streptomyces* species. In this context, it should be noted that evidence has been presented for niche varieties between *S. griseus* strains [55].

It is evident from the 16S rRNA gene sequence data that many of the *Micromonospora* isolates active in the anti-tumor and PTP1B assays belong to putatively new species of this genus. Indeed, this also applies to isolates assigned to some of other taxa, such as the genera *Rhodococcus, Nomomuraea and Verrucosispora,* thereby indicating that mangrove habitats are likely to be a good source of rare actinomycetes. Taxonomic descriptions of new species discovered in this study will be the subject of subsequent publications. At present, detailed conclusions regarding the distribution and ecology of actinomycete taxa in mangrove habitats is premature. However, it is clear from the present study that actinomycetes isolated from mangrove habitats can be expected to provide high quality biological material for high through put biochemical, anti-cancer and anti-infection screening programmes.

## 4. Experimental Section

### 4.1. Collection of samples

**Figure 4:**
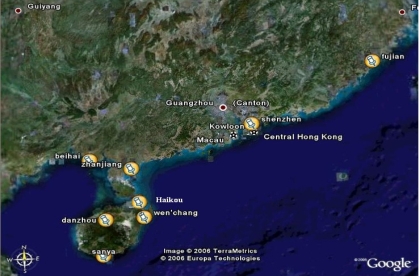
Location of sampling sites.

Soil samples were collected from mangrove sites at Danzhou, Haikou, Sanya and Wenchang in Hainan Province; Shenzhen and Zhanjiang in Guangdong Province; Xiameng in Fujian Province, and Beihai in Guangxi Province (Figure 4). At each location, five soil core samples were collected at a depth of 0–30 cm within a 100 m^2^ area. The soils from each location were bulked and homogenized to prepare composite samples. In addition, leaf, stem, roots, and where relevant flowers and fruits, were collected together with rhizophere soil from plant species growing in the mangrove forest at Wenchang, Hainan Province (Table 5). The soil and plant tissue samples were either examined on the day of sampling or on the following day. Samples were stored at 4 °C in the dark prior to examination.

### 4.2. Selective isolation of actinomycetes from environmental samples

Air dried soil samples were sieved to exclude large mineral and organic matter particles then ground in a pestle and mortar. Selective pretreatments of the soil samples included dry heat (120 °C, 60 min) [56], treatments with Chloramine-T [57]; phenol (1.5 %, 30 min at 30 °C) [56]; 0.05 % SDS and 6 % yeast extract (40 °C, 200 rpm, 30 min) [56], and wet heat in sterilized sea water (50 °C, 15 min) [58]. The pretreated soil samples were diluted 1:10 v/v with sterile 1/4 Ringer’s solution and serial dilutions prepared down to 10^–4^. One hundred μL of the 10^–1^ −10^−4^ suspensions were spread, in triplicate, onto selective isolation media.

The plant tissues were surface cleaned with sterile water and air-dried in a laminar flow hood. The resultant preparations were treated with 75% ethanol for 5 min, then with 0.1% HgCl_2_ for 15 min, prior to washing five times for 5 minutes with sterile 1% Tween 80. Five grams of surface sterilized mangrove plant tissues were cut into small pieces, added to 45 mL 50% sterile sea water, and ground into smaller pieces using a pestle and mortar. Fractions (100 μL) of the resultant preparations were plated, in triplicate, onto selective isolation plates which had been dried for 30 minutes at room temperature in a laminar flow cabinet.

Dilutions of soil and plant tissues were plated out onto one or more of 11 different selective isolation media (IM1 to IM11):IM1 (oatmeal agar, ISP meduim3) [59]; oatmeal, 60 g; agar, 15 g; pH 7.2~7.4; IM2 (Gause modified medium 1 [60]); soluble starch, 20 g; K_2_HPO_4_, 0.5 g; KNO_3_, 1 g; MgSO_4_·7H_2_O, 0.5 g; agar, 20 g; pH7.4~7.6; IM3 (Raffinose-histidine medium[61]); raffinose, 10 g; L-histidine, 1g; MgSO_4_·7H_2_O, 0.5 g; FeSO_4_·7H_2_O, 0.01 g; K_2_HPO_4_, 1g; Bacto-agar, 20.0 g; pH 7.0~7.4; IM4, 1/10 ATCC 172 medium; glucose, 1 g; soluble starch, 2 g; yeast extract, 0.5 g; CaCO_3_, 1.5g; agar, 18 g; N-Z-amine, 0.5 g; pH 7.0~7.4, and supplemented with a filter sterilized solution of novobiocin (25 mg/ml); IM5, sea-water agar; sea-water, 1L; agar 18 g; IM6 (Yeast extract-malt estract agar; ISP medium 2[59]); yeast extract, 4 g; malt extract, 30 g; glucose, 4 g; agar, 18 g; pH 7.0~7.4; IM7 (Starch casein agar [62]); starch, 10 g; casein, 3 g; agar, 18 g; pH 7.0~7.4; IM8 [63]; glucose, 10 g; peptone, 5 g; tryptone, 3 g; NaCl, 5 g; agar, 15 g; pH 7.0, and supplemented with filter sterilized nalidixic acid (10 mg/mL) and novobiocin (10 mg/mL); IM9 (Glucose asparagine agar [58]); glucose, 10g; asparagine, 0.5 g; K_2_HPO_4_, 0. 5 g; agar, 20 g; pH: 7.2~7.4; IM10 (humic acid -vitamin agar [64]); humic acid, 1.0 g; CaCO_3_, 0.02 g; Na_2_HPO_4_, 0.5 g; KC1, 1.7 g; FeSO_4_.7H_2_O, 0.01 g; MgSO_4_·7H_2_O, 0.5 g; agar, 18 g; pH7.2, and supplemented with sterilized vitamins: aminobenzoic acid, inositol, nicotinic acid, riboflavin, pantothenic acid, thiamine, vitamin B6 (at 0.5mg) and 0.25 mg of biotin; IM11 (dextran-histidine-sodium chloride-mineral salts agar[65]); K_2_HPO_4_, 2 g; KNO_3_, 2 g; MgSO_4_·7H_2_O, 0.05 g; CaCO_3_, 0.02 g; FeSO_4_·7H_2_O, 0. 01 g; agar, 18 g; distilled water, 1 L; pH 7.2, and supplemented with dextran, 1 %,w/v; <sc>l</sc>-histidine, 0.1 %, w/v; NaCl, 3 %, w/v; penicillin G (5 iu mL^–1^), and rifampicin (5 μg/mL). All of the media, apart from IM5, were supplemented with 50–100 mg/L K_2_Cr_2_O_7_, 50 mg/L cycloheximide and 25–50 mg/L nystatin [66], and made up to one liter with 0.5 L sea-water and 0.5 L dH_2_O.

The inoculated plates were incubated at 28 °C for one up to twelve weeks. Colonies of streptomycete-like and non-streptomycete-like strains growing on the isolation plates were inoculated onto IM2 and IM6 plates, respectively, and incubated for 7–10 days at 28 °C. Purified cultures were stored either on IM 4 or IM6 agar slants for short term storage and in 20% glycerol at −80 °C for long-term storage.

### 4.3. Preparation of crude extracts

The fermentation medium for primary screening was FM 3 [67]: soluble starch, 20 g; soy powder, 15 g; yeast extract, 5 g; peptone, 2 g; CaCO_3_, 4 g; sea salts, 18 g in 1 L distilled water, pH 7.2~7.4. Two media were used for re-screening, namely FM 2 [68]; Glucose, 10 g; soluble starch, 30 g; yeast powder, 2 g; casein, 4 g; K_2_SO_4_, 8 g; MOPS (3-[*N*-morpholino]propanesulfonic acid), 5 g; sea salts, 18 g; distilled water, 1 L; pH 7.2–7.4; and FM 17 [69]; Glucose, 20 g; yeast powder, 5 g; casein, 5 g; KNO_3_, 15 g; CaCO_3_, 4 g; sea salts, 18 g; distilled water, 1 L; pH 7.2–7.4. All three media were autoclaved at 121 °C for 20 min.

Each of 2,041 purified isolates were transferred to a test tube (30 mm×200 mm) which contained 20 mL of the relevant fermentation medium, and cultured at 200 rpm, at an angle of 45º, for 7–10 days at 28 °C. Crude extracts were prepared by adding 60 mL of methanol to each of the cultures and the extraction allowed to proceed for 2 weeks. One mL fractions of the resultant extracts were transferred to wells in deep 96-well plates, vacuum dried at 60 °C, dissolved in 200 μL DMSO and used in the biochemical screens and for cytotoxicity assay against HCT-116 cells.

### 4.4. Biological assays

#### Antimicrobial activity

The method used was modified from that of Hong and Xiao [70]. *C. albicans* ATCC 10231 and *S. aureus* ATCC 51650 were cultured overnight at 30 °C at 200 rpm in YPD medium (glucose, 2%; tryptone, 2%; yeast extract, 1%; pH 5.0~5.5) and nutrient broth, respectively. The resultant cultures were diluted in the same media to 0.8–1.2×10^6^ CFU/mL, and 100 μL aliquots of this inoculum were transferred to individual wells in a 96-well plates. Aliquots (100 μL) of the fermentation broth supernatant of each of the isolates (fermented in the media above mentioned, and centrifuged at 10,000 rpm at 4 °C) were added to each of the wells. The two culture media were used as negative controls, and fluconazole and kanamycin as the positive controls for the anti- *C. albicans* and anti *S. aureus* assays, respectively. The 96-well plates were shaken at 200 rpm for 24h at 30 °C  then assayed using a microplate spectrophotometer (Multiskan Mk3, Finland) at 570 nm. The absorbance readings from each well were used to record bioactivities as “+”, “++”, or “+++” for higher or equal to 4–6, 6–8 and 8 μg/mL of standard, respectively. Serial dilutions of the antibiotics (128, 64, 32, 16, 14, 12, 10, 8, 6, 4, 2 and 1 μg/mL) were used to generate standard curves.

#### Cell growth inhibition assay

Fractions (100 μL) of each cell suspension (30,000 cells/mL) of the adherent cell line HCT-116 were dispensed into individual wells in 96-well plates which were incubated in McCoy's 5A medium (Sigma-Aldrich Corp., St. Louis, MO) at 37 °C in an incubator containing 5% CO_2_ for 24 hours to allow surface attachment of the cells. Preparation in DMSO (1 μL) were added and the 96-well plates incubated under the same conditions for 72 hours when 30 μL MTT (5 mg/mL) was added to each of the wells and the plates incubated for 3 hours under the same conditions. The media were removed using a pipette and 100 μL DMSO added to each of the wells of the plates. Readings were taken using a SpectraMAX340 microplate reader (Molecular Devices, Sunnyvale, CA) at an absorbance of 550 nm, and a reference wavelength at 690 nm. Adriamycin was used as the positive control. The IC_50_ value of each inhibitor was calculated in GraphPad Prism (GraphPad Software, San Diego, CA) using non-linear regression analysis and a sigmoidal dose response (variable slope) equation. Data were presented as mean ± S.D. of ≥3 independent experiments unless.

#### Biochemical assays

The construction, expression, purification, and enzymatic assays for protein tyrosine phosphatase 1B (PTP1B) and caspase 3 were carried following Shi [71] and Du [72], respectively. Aurora a [73], a recombinant protein kinase domain (125–391 amino acids according to AAH02499) was expressed in an *Escherichia coli* system; determination of the enzymatic activity was achieved using a Z-LYTE kinase assay kit (Invitrogen, Carlsbad, CA ). For primary screening, 2 μL of the stock solution of each crude extract (1 mg/mL) in DMSO were transferred into individual wells of 96-well flat bottom plates to give a final concentration of 20 μg/mL of extract in 2% DMSO. After incubation with the enzymes for 15 min, 10 times concentrated substrates were added to initiate the enzymatic reaction, and the resultant enzymatic activity normalized against the control (2% DMSO) to obtain the inhibition rate of the compound. When the inhibition rate was more than 50% at 20 μg/mL, the dose-response inhibition assay of the compound was performed to determine the 50% percentage inhibition concentrations (IC_50_).

### 4.5 Identification of isolates

#### Morphological characterization

The presumptive streptomycetes were inoculated onto oatmeal agar plates [62] prepared with and without sea salts, and onto peptone-yeast extract-iron agar [59] plates and incubated at 28 °C for 3 weeks and 4 days, respectively. After incubation, the oatmeal agar plates were examined by eye to record aerial spore mass color, substrate mycelial pigmentation, and the color of any diffusible pigments. The peptone-yeast extract-iron agar plates were examined for the production of melanin pigments

#### Chemotaxonomy

Biomass was harvested from isolates grown at 28 °C for 3–7 days on IM4 or IM6 agar plates for streptomycte-like and non-streptomycete-like strains, respectively. Isomers of diaminopimelic acid and diagnostic whole-organism sugars were detected by using the procedures described by Hasegawa *et al.* [74].

#### Extraction of DNA from pure cultures and PCR amplification

Total genomic DNA samples from 243 representative strains were extracted with the Fast DNA®SPIN Kit used for extracting community DNA from soil (Q-BIOgene, USA) using a protocol modified from that of the manufacturer ( Goodfellow, 2007). In short, approximately 0.5g of each culture was suspended in TE buffer (0.5ml) and ribolised for 30s at a speed of 5.5 m/s following the addition of sterile glass beads (0.5g, 100 mesh). The resultant preparations were extracted with an equal volume of chloroform: *iso*-amyl alcohol (24:1, v/v) and centrifuged at 15,000g for 5min at 4 □. The upper aqueous layers, which contained the DNA, were transferred to fresh tubes and used as template DNA. The PCR reactions were performed in a final volume of 25μl which was composed of template DNA (1μl upper aqueous layer), 1.5mM MgCl_2_, 0.2mM of each dNTP, 200pM of primer Eubac27F and primer Eubac1492R[75], and 1U of *Taq* polymerase with the appropriate reaction buffer under the following conditions: initial denaturation at 95 °C for 5 min, followed by 28 cycles of 95 °C for 50 s, annealing at 52 °C for 50s, and 72 °C for 90s. The amplification products were separated by gel electrophoresis in 1% agarose gels which were stained with Goldview^TM^ Nucleic Acid Stain (SBC, China).

#### Cloning, sequencing and phylogenetic analyses

The PCR products were blunt-end ligated to the plasmid vector pEGM (Promega, USA) and the ligation products transformed into competent *E. coli* DH5α cells. The resultant clones were sequenced using an ABI 3730 Automated Sequencer (Applied Biosystems), and the sequences submitted to the BLAST function of GenBank. The cloned 16S rRNA gene sequences were aligned using CLUSTAL-X software [76] in the program BioEdit [77]. A neighbor joining [78] phylogenetic tree was generated using MEGA version 4.0 software [79], was evaluated in a bootstrap analysis [80] of 1,000 replicates; a distance matrix was generated using Kimura’s 2-parameter model [81].

## Figures and Tables

**Table 1:** Selective isolation of actinomycetes from mangrove environmental samples.

	Number of samples / Number of isolated actinomycetes			
Sites	Composite soil	Rizhosphere soil	Plant tissues	Total
Danzhou	5/40	ND	ND	5/40
Fujian	3/93	ND	ND	3/93
Guangxi	17/62	5/27	4/4	26/93
Haikou	3/25	5/16	2/11	10/52
Sanya	3/78	ND	ND	3/78
Shenzhen	4/57	5/44	8/48	17/149
Wenchang	26/205	31/917	62/256	119/1378
Zhanjiang	5/43	ND	23/115	28/158
Total	66/603	46/1004	99/434	211/2041

**Table 2:** Percentage of bioactive strains isolated from plant tissues against those isolated from corresponding rhizosphere soil.

Sampling sites	Sample type	Anti-*C. albicans*	Anti-*S. aureus*	Anti-tumor cell	PTP1B inhibition
Wenchang	Plant tissues	9.8	16.0	22.2	1.6
	Rhizosphere soil	3.5	8.2	14.4	1.2
Zhangjiang	Plant tissues	20.0	37.4	4.3	/
	Rhizosphere soil	15.0	2.3	25.0	5.0

**Table 3:** Number of bioactive actinomycetes isolated from rhizosphere soils and plant tissues of 23 mangrove plant species.

	Number of Bioactive actinomycetes							
	Rhizosphere soil				Plant tissues			
	
Mangrove plant species	Anti-*C. albicans*	Anti-*S. aureus*	Anti- tumor cell	PTP1B inhibition	Anti-*C. albicans*	Anti-*S. aureus*	Anti- tumor cell	PTP1B inhibition
*Acanthus ilicifolius*	1	5	13	2	6	3	5	3

*Acrostichum aureum*	0	0	0	0	2	1	0	0

*Acrostichum speciosum*	0	2	7	0	0	2	2	0

*Aegiceras corniculatum*	3	15	5	0	5	11	10	0

*Avicennia marina*	1	0	0	0	4	7	1	0

*Barringtonia racemosa*	0	0	1	0	ND	ND	ND	ND

*Bruguiera gymnorrhiza*	2	4	5	0	12	15	6	1

*Bruguiera sexangula*	1	2	12	2	0	0	1	0

*Cerbera manghas*	5	0	4	3	1	4	4	0

*Ceriops tagal*	0	0	0	0	2	1	1	0

*Excoecaria agallocha*	0	0	3	0	0	3	2	0

*Heritiera littoralis*	13	26	22	2	3	3	2	0

*Hibiscus tilisaceus*	6	16	33	4	7	4	7	0

*Kandelia candel*	0	0	1	0	2	4	1	0

*Lumnitzera racemosa*	0	0	0	0	0	2	2	1

*Pongamia pinnata*	0	7	6	0	0	2	2	0

*Rhizophora apiculata*	0	2	11	3	0	1	2	0

*Rhizophora stylosa*	ND	ND	ND	ND	7	14	3	0

*Sonneratia alba*	7	12	10	0	0	2	3	0

*Sonneratia caseolaris*	1	4	6	1	0	3	4	0

*Sonneratia hainanensis*	0	0	0	0	0	0	0	0

*Sonneratia paracaseolaris*	2	4	5	0	0	4	1	0

*Xylocarpus granatum*	0	0	0	0	4	2	3	0

Total	42	99	144	17	55	88	62	5

**Table 4:** Identification of representative bioactive actinomycetes isolated from Chinese mangroves.

Genera	Families	Suborders	Number of the identified representative strains				
			Anti-*C. albicans*	Anti-*S. aureus*	Anti-tumor cell	Caspase 3 inhibition	PTP1B- inhibition
*Actinomadura*	*Thermomonosporaceae*	*Streptosporangineae*			3		

*Microbispora*						1	

*Nonomuraea*	*Streptosporangiaceae*				1		

*Actinoplanes*	*Micromonosporaceae*	*Micromonosporineae*	1				

*Micromonospora*			7	10	25	1	8

*Verrucosispora*					1		

*Arthrobacter*	*Micrococcaceae*	*Micrococcineae*		1			

*Isoptericola*			1				

*Micrococcus*			1				

*Microbacterium*	*Microbacteriaceae*		1				

*Nocardia*	*Nocardiaceae*	*Corynebacterineae*			5		

*Rhodococcus*				1	1		

*Strepomyces*	*Streptomycetaceae*	*Streptomycineae*	1	7	45	1	4

**Table 5:** Source of plant materials and soils collected from the mangrove forest at Wenchang, Hainan Province.

Genus and Species	Samples
*Acanthus ilicifolius*	Rhizosphere soil, roots, leaves and flowers
*Acrostichum aureum*	}Rhizosphere soil, roots and leaves
*Acrostichum speciosum*	
*Aegiceras corniculatum*	
*Avicennia marina*	Rhizosphere soil, leaves, and fruit
*Barringtonia racemosa*	Rhizosphere soil
*Bruguiera gymnorrhiza*	}Rhizosphere soil, roots and leaves
*Bruguiera sexangula*	
*Cerbera manghas*	}Rhizosphere soil, roots, leaves, and fruit
*Ceriops tagal*	
*Excoecaria agallocha*	Rhizosphere soil, roots and leaves
*Heritiera littoralis*	Rhizosphere soil, leaves, and fruit
*Hibiscus tilisaceus*	Rhizosphere soil, roots and leaves
*Kandelia candel*	Rhizosphere soil and leaves
*Lumnitzera racemosa*	Rhizosphere soil, roots and leaves
*Pongamia pinnata*	Rhizosphere soil and leaves
*Rhizophora apiculata*	Rhizosphere soil and leaves,
*Rhizophora stylosa*	Roots and leaves
*Sonneratia alba*	}Rhizosphere soil, leaves, and fruit
*Sonneratia caseolaris*	
*Sonneratia hainanensis*	Rhizosphere soil and leaves
*Sonneratia paracaseolaris*	Roots and leaves
*Xylocarpus granatum*	Roots, leaves, and fruit
