# Correction: Hong, K. *et al*. Actinomycetes for Marine Drug Discovery Isolated from Mangrove Soils and Plants in China. *Mar. Drugs* 2009, *7*, 24–44

**DOI:** 10.3390/md7040495

**Published:** 2009-10-21

**Authors:** Kui Hong, An-Hui Gao, Qing-Yi Xie, Hao Gao, Ling Zhuang, Hai-Peng Lin, Hai-Ping Yu, Jia Li, Xin-Sheng Yao, Michael Goodfellow, Ji-Sheng Ruan

**Affiliations:** 1 Institute of Tropical Bioscience and Biotechnology, Chinese Academy of Tropical Agriculture Sciences, Haikou 571101, China; E-Mails: xie-qy@sohu.com (Q.-Y.X.); linhp010612@gmail.com (H.-P.L.); 2 National Center for Drug Screening, Shanghai Institute of Materia Medica, Shanghai 201203, China; E-Mails: ahgao@mail.shcnc.ac.cn (A.H.G.); hpyu@mail.shcnc.ac.cn (H.-P.Y.); jli@mail.shcnc.ac.cn (J.L.); 3 Institute of Traditional Chinese Medicine & Natural Products, College of Pharmacy, Jinan University, Guangzhou 510632, China; E-Mails: ghao@mail.tsinghua.edu.cn (H.G.); tyaoxs@jnu.edu.cn (X.-S.Y.); 4 University of Newcastle, Newcastle upon Tyne NE1 7RU, UK; E-Mail: m.goodfellow@ncl.ac.uk (M.G.); 5 Institute of Microbiology, Chinese Academy of Sciences, Beijing, 100081, China; E-Mail: jishengruan@yahoo.com.cn (J.-S.R.)

We found an error in our paper published in Marine Drugs [1]. Two words have been lost by mistake at the end of one sentence on page 38. Thus, the last sentence of the paragraph of “Cell growth inhibition assay” should be “Data were presented as mean ± S.D. of ≥3 independent experiments unless otherwise specified.”

We apologize for any inconvenience caused to the readers.
